# A single residue in the αB helix of the E protein is critical for Zika virus thermostability

**DOI:** 10.1038/s41426-017-0006-9

**Published:** 2018-01-24

**Authors:** Dong-Yang Xie, Zhong-Yu Liu, Qing-Gong Nian, Ling Zhu, Nan Wang, Yong-Qiang Deng, Hui Zhao, Xue Ji, Xiao-Feng Li, Xiangxi Wang, Pei-Yong Shi, Cheng-Feng Qin

**Affiliations:** 1 Department of Virology, State Key Laboratory of Pathogen and Biosecurity, Beijing Institute of Microbiology and Epidemiology, Beijing, 100071 China; 2 Anhui Medical University, Hefei, 230032 China; 3 National Laboratory of Macromolecules, Institute of Biophysics, Chinese Academy of Science, Beijing, 100101 China; 4 Department of Biochemistry and Molecular Biology, Department of Pharmacology and Toxicology, Sealy Center for Structural Biology & Molecular Biophysics, University of Texas Medical Branch, Galveston, TX 77555 USA

Zika virus (ZIKV) has been linked to a panel of unexpected biological features that diverge significantly from other well-known mosquito-borne flaviviruses, such as dengue virus (DENV), yellow fever virus (YFV), Japanese encephalitis virus (JEV), and St. Louis encephalitis virus (SLEV). A recent study showed that ZIKV displayed much greater stability than DENV at high temperatures, leading to the hypothesis that this superior stability of ZIKV may contribute to its unique pathology and routes of transmission^1^.

One interesting feature of viral thermostability is that the variations often have definite genetic and structural basis^2^. A cryo-electron microscopy structural reconstruction of ZIKV virions revealed two distinct structural features of ZIKV: (i) an extended glycan loop structure and (ii) an extra hydrogen-bonding interaction between residues Q350 and T351 of domain III around the 5-fold vertex, which might contribute to the observed high stability of ZIKV^1, 3^. However, follow-up investigations using replicon-based virus-like particle (VLP) and infectious clones have concluded that these domains and interactions are not related to the thermostability of ZIKV^4, 5^.

Interestingly, a comparison of the thermostabilities of ZIKV/DENV chimeric viruses with their parental viruses clearly demonstrated that the predominant determinants of ZIKV thermostability lie in the prM-E proteins^4^. This result inspired us to further explore the potential molecular determinants within the prM-E proteins by using the reverse genetics system of ZIKV^6^. Recently, we demonstrated the critical role of residue 264 of the E protein (E-264) in modulating the stability of JEV, and a single H264Q substitution in the E protein significantly decreased the thermal stability of JEV^7^. Residue 264 is located in the αB helix of domain II of the E protein (residues 259–266) of JEV. Sequence alignment revealed that the homologous residue E-264 is threonine 267 (T267) in ZIKV, which is highly conserved among most flaviviruses, including YFV, DENV and SLEV (Fig. 1a), and among all ZIKV strains (Figure S1). To verify the potential role of this residue in ZIKV thermal stability, we introduced the T267Q and T267H mutations (the primer sets used in this study are listed in Table S1) into the αB helix of the E protein based on the infectious clone of ZIKV^6^. Both mutant viruses were successfully constructed and recovered in BHK-21 cells with similar peak titers and plaque morphologies to the WT ZIKV (Fig. 1b). Standard thermal stability assays were performed as previously described^1^, and the results demonstrated that the ZIKV/T267H mutant virus showed the same resistance to heat as the WT ZIKV at 50 °C. Meanwhile, the ZIKV/T267Q mutant was highly sensitive to heat treatment, and incubation for 30 min completely abolished ZIKV infectivity (Fig. 1c).

**Fig. 1:**
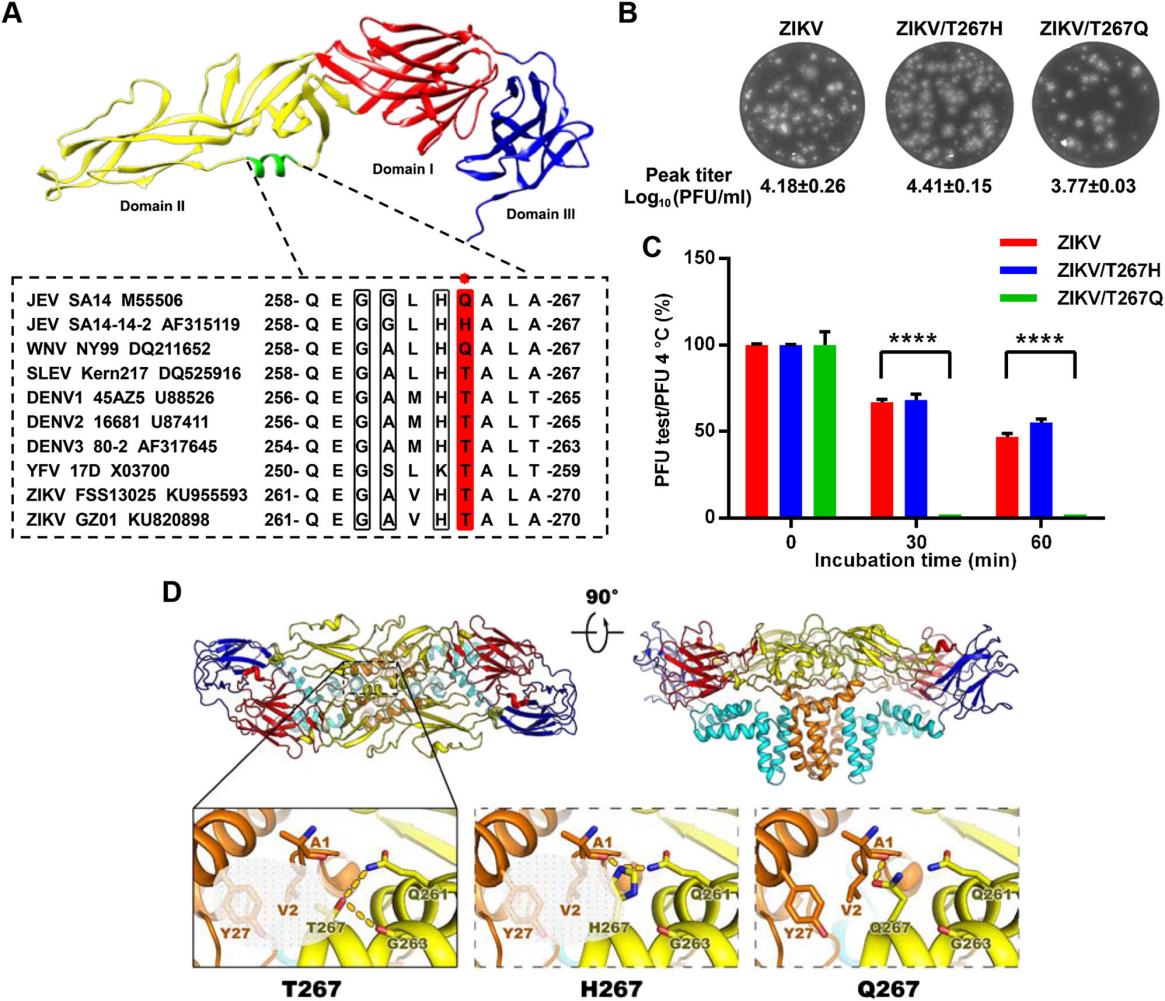
Characterization of critical residues in the αB helix of the ZIKV E protein **a** Upper panel: crystal structure of the JEV E monomer with the αB helix highlighted in green (PDB: 3P54); lower panel: sequence alignment of the αB helices from the E proteins of different flaviviruses. The E-264 residue (numbering based on JEV) is highlighted in red. The other three conserved residues adjacent to E-264 are shown in gray. **b** Plaque morphologies and peak titers (*N *= 2) of ZIKV and the T267 mutants. **c** Thermostability analysis of ZIKV and the T267 mutants at 50 °C. ****P < 0.0001. *N* = 2. A one-way analysis of variance (ANOVA) was performed to analyze the significant differences between each treatment group and the corresponding untreated group, and the data were expressed as the mean ± standard deviation. **d** A potential interaction network centering on T267/H267/Q267 near helix αB was analyzed using the SWISS-MODEL Workspace in the Expasy web server. Top: side view of the atomic model of the E:M:M:E heterotetramer of ZIKV (PDB code: 5IRE) shown in ribbon. Domains I, II, III, and the transmembrane (TM) of E and M are shown in red, yellow, blue, cyan, and orange, respectively. Bottom: the detailed interaction networks centering on residue 267 from structures of the WT ZIKV and the T267 mutants. Structural figures were prepared with PyMol and UCSF Chimera version 1.10.1 (the Regents of the University of California)

A structural analysis of the E:M:M:E heterotetramer of ZIKV revealed that residue T267 within helix αB of ZIKV is capable of stabilizing viral particles through both hydrophobic and hydrophilic interactions. Residue T267 interacts with V2 and Y27 from the M protein through hydrophobic interactions and forms hydrogen bonds with Q261 and G263 of the E protein, and H267 forms hydrogen bonds with A1 from the M protein and Q261 from the E protein (Fig. 1d, Supplementary Table S2). However, the replacement of H267 or T267 with Q267 disrupts the hydrophobic interactions with the E or M proteins and only maintains weak hydrogen bonds with A1 from the M protein (Fig. 1d and Supplementary Table S2). This finding suggests that the hydrophobic and hydrophilic interactions between the αB helix of the E and M proteins are critical for flavivirus thermostability.

In this report, we identified a highly conserved residue in the αB helix in the E protein of ZIKV as a potent molecular determinant for its thermal stability. These findings will not only help us understand the molecular and structural basis of flavivirus stability, but they also provide important clues for the rational design of a flavivirus vaccine with improved thermostability^8^.

## Electronic supplementary material

Supplementary Figure S1

Supplementary Table S1

Supplementary Table S2
