# Supplementary figures and images for: A single residue in the αB helix of the E protein is critical for Zika virus thermostability
Source: Emerg Microbes Infect. 2018 Jan 24;7:5. doi: 10.1038/s41426-017-0006-9 (PMC5837149; doi:10.1038/s41426-017-0006-9)

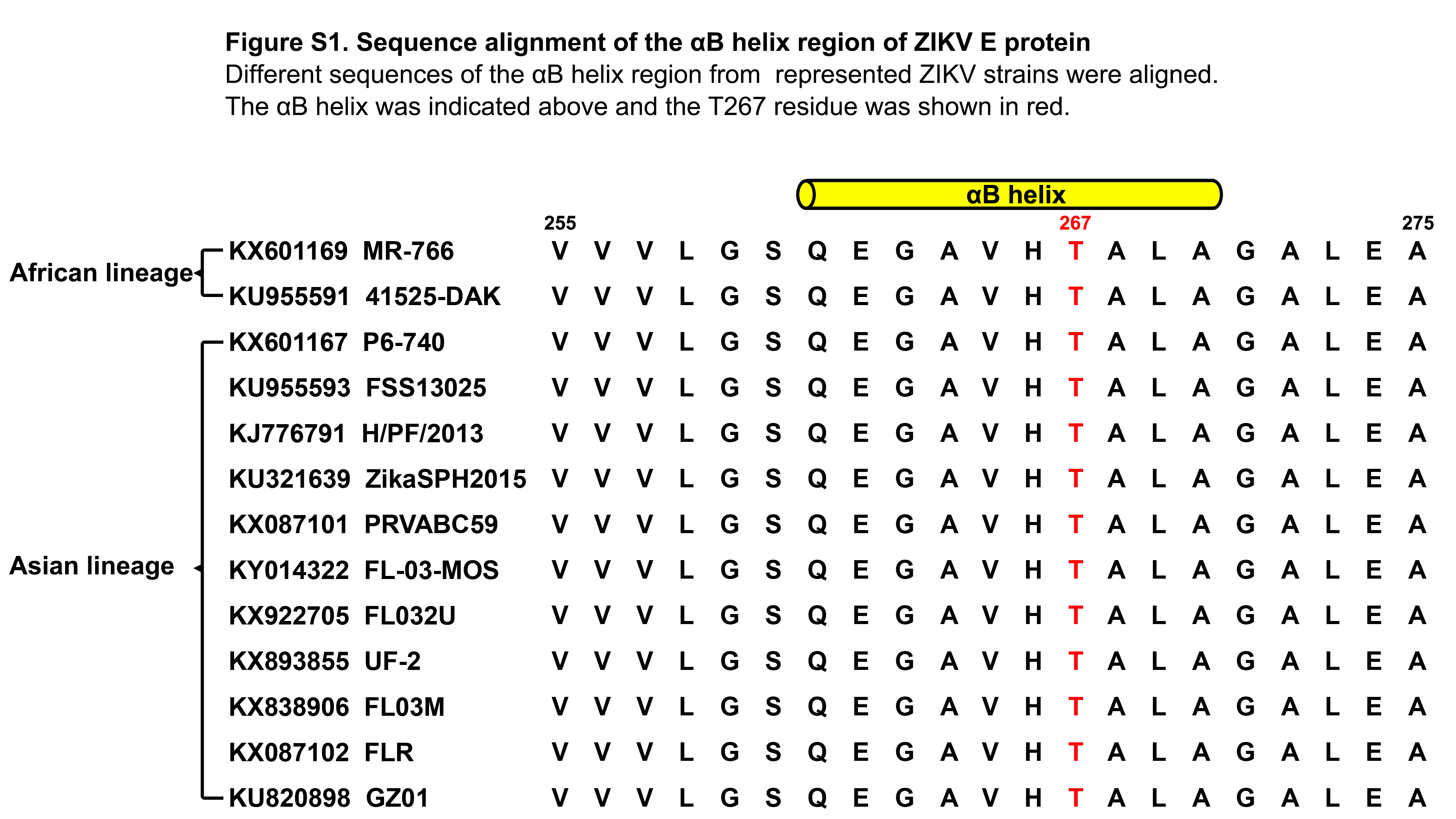

Supplement: Supplementary file 1 — Supplementary Figure S1 [file 41426_2017_6_MOESM1_ESM.jpg]
