# Supplementary material for: A single residue in the αB helix of the E protein is critical for Zika virus thermostability
Source: Emerg Microbes Infect. 2018 Jan 24;7:5. doi: 10.1038/s41426-017-0006-9 (PMC5837149; doi:10.1038/s41426-017-0006-9)
Supplement: Supplementary file 2 — Supplementary Table S1 [file 41426_2017_6_MOESM2_ESM.docx]

**Table S1. Primers used for reverse genetics of ZIKV and YFV**

| **Primer** | **Sequence (5′-3′)** | **Position*** |
| --- | --- | --- |
| **ZIKV-Avr II-F** | **TGGAAGCCTAGGACTTGATTGTG** | **1526-1548** |
| **ZIKV-Pml I-R** | **TTCCTCCACGTGGACCTTAGTG** | **3335-3356** |
| **ZIKV-T267Q-F** | **CAGTTCATCAGGCCCTTGCTGG** | **1768-1789** |
| **ZIKV-T267Q-R** | **AGGGCCTGATGAACTGCTCCTTCTTG** | **1758-1783** |
| **ZIKV-T267H-F** | **CAGTTCATCATGCCCTTGCTGG** | **1768-1789** |
| **ZIKV-T267H-R** | **AGGGCATGATGAACTGCTCCTTCTTG** | **1758-1783** |

* The primer binding positions were based on ZIKV strain FSS13025 (KU955593).
