# Supplementary material for: A single residue in the αB helix of the E protein is critical for Zika virus thermostability
Source: Emerg Microbes Infect. 2018 Jan 24;7:5. doi: 10.1038/s41426-017-0006-9 (PMC5837149; doi:10.1038/s41426-017-0006-9)
Supplement: Supplementary file 3 — Supplementary Table S2 [file 41426_2017_6_MOESM3_ESM.docx]

**Table S2. Interactions between the E-267 residue within helix αB and its environment**

| Residue | Interaction type | Atom | | Distance | Estimated Strength# |
| --- | --- | --- | --- | --- | --- |
| T267 | Hydrogen bonds | T267 (OG1) | Q261 (NE2) | 2.9 Å | -3.2 kcal/mol |
|  |  | T267 (CG2) | G263 (O) | 3.1 Å | -2.9 kcal/mol |
|  | Hydrophobic interactions | T267 (CG2) | A1*, V2*, Y27* |  | -4.5 kcal/mol |
| H267 | Hydrogen bonds | H267 (NE2) | A1* (O) | 2.7 Å | -3.3 kcal/mol |
|  |  | H267 (NE2) | Q261 (NE2) | 2.9 Å | -3.0 kcal/mol |
|  | Hydrophobic interactions | H267 (CO2, CG) | A1*, V2*, Y27* |  | -4.8 kcal/mol |
| Q267 | Hydrogen bond | Q267 | A1* (O) | 3.1 Å | -2.9 kcal/mol |

The symbol * represents the residue from M protein.

#The distance of strengths of hydrogen bonds and hydrophobic interactions were estimated by PISA.
